# Congenital Laryngeal Stridor

**Published:** 1903-08-15

**Authors:** 


### CONGENITAL LARYNGEAL STRIDOR.

In a paper dealing with this rare condition Dr. Newcomb[1] defines the term as meaning a stridor coming on within a few days of birth, continuing for a certain period, measured usually in months, but possibly in years, apparently not doing the child much if any injury, and finally subsiding of its own accord without sequelæ. No treatment has seemed to shorten or modify the course of the affection, and the literature on the subject has mainly concerned itself with theories of etiology and criteria of differential diagnosis. Where laryngeal examination has been applicable it has revealed a malformation of the vestibule, epiglottis or ary-epiglottic folds. The epiglottis has been found folded on itself, and the ary-epiglottic folds have been devoid of their usual firmness, so that their free ends, being thin and flabby, have nearly touched. In other cases the larynx has appeared to be normal. The causes of the condition are obscure. Some have regarded the stridor as merely a modification of the nervous mechanism of the larynx which is within the limits of health, and in most of the recorded cases the general health has been good. In fact the disorder has been looked upon as more an annoyance to others than to the child itself. The time of onset may be any period from the first day of life. In the 18 cases reported by Sutherland and Lack the onset occurred on the first day in 11. These authors believe it rare to have the onset delayed after the second week. The character of the sound made varies considerably in different cases; it may be a stridor, a croak, a cluck, etc. With superficial breathing it may intermit, to become continuous again when the breathing is deep. It is increased by changes of temperature, excitement, sudden waking, coughing and flatulency. It sometimes stops when the position of the child is changed from the back to one side, or when the head is placed at a lower level than the body. It continues during sleep, though never aggravated thereby, and persists during chloroform anæsthesia. With regard to prognosis, in weakly children the condition may lead to thoracic distortion and may prove fatal. As a rule it passes off by the end of the second year. It may return during periods of excitement or of ill-health. It generally increases in severity for some weeks after its appearance, then becomes stationary until about the eighth or ninth month, and then gradually diminishes until a period from the eighteenth to the twenty-fourth month, when it disappears. The condition must not be confused with laryngismus stridulus. The latter comes on later, often with the first dentition. The patients are rickety and may have tetany, convulsions or other nerve disturbance. The spasms are of long duration, and the patients become cyanotic. Crying may set the spasm up or aggravate it, and attacks are more frequent during night hours. The treatment of congenital laryngeal stridor consists merely of general tonics as no special treatment has been found to modify the course of the disorder.

[1] Medical Record, July 25.

